# Dynamic nomograms combining N classification with ratio-based nodal classifications to predict long-term survival for patients with lung adenocarcinoma after surgery: a SEER population-based study

**DOI:** 10.1186/s12885-021-08410-6

**Published:** 2021-08-04

**Authors:** Suyu Wang, Yue Yu, Wenting Xu, Xin Lv, Yufeng Zhang, Meiyun Liu

**Affiliations:** 1 Department of Cardiothoracic Surgery, Changzheng Hospital, Naval Medical University, 415 Fengyang Road, Shanghai, 200003 China; 2 Fuyang Hospital of Anhui Medical University, 99 Huangshan Road, Fuyang, China; 3 Department of Anesthesiology, Shanghai Pulmonary Hospital, Tongji University School of Medicine, 507 Zhengmin Road, Shanghai, 200433 China

**Keywords:** Nomogram, Log odds of positive lymph nodes (LODDS), Lymph node ratio (LNR), Lung adenocarcinoma, SEER program

## Abstract

**Background:**

The prognostic roles of three lymph node classifications, number of positive lymph nodes (NPLN), log odds of positive lymph nodes (LODDS), and lymph node ratio (LNR) in lung adenocarcinoma are unclear. We aim to find the classification with the strongest predictive power and combine it with the American Joint Committee on Cancer (AJCC) 8th TNM stage to establish an optimal prognostic nomogram.

**Methods:**

25,005 patients with T1-4N0–2M0 lung adenocarcinoma after surgery between 2004 to 2016 from the Surveillance, Epidemiology, and End Results database were included. The study cohort was divided into training cohort (13,551 patients) and external validation cohort (11,454 patients) according to different geographic region. Univariate and multivariate Cox regression analyses were performed on the training cohort to evaluate the predictive performance of NPLN (Model 1), LODDS (Model 2), LNR (Model 3) or LODDS+LNR (Model 4) respectively for cancer-specific survival and overall survival. Likelihood-ratio χ^2^ test, Akaike Information Criterion, Harrell concordance index, integrated discrimination improvement (IDI) and net reclassification improvement (NRI) were used to evaluate the predictive performance of the models. Nomograms were established according to the optimal models. They’re put into internal validation using bootstrapping technique and external validation using calibration curves. Nomograms were compared with AJCC 8th TNM stage using decision curve analysis.

**Results:**

NPLN, LODDS and LNR were independent prognostic factors for cancer-specific survival and overall survival. LODDS+LNR (Model 4) demonstrated the highest Likelihood-ratio χ^2^ test, highest Harrell concordance index, and lowest Akaike Information Criterion, and IDI and NRI values suggested Model 4 had better prediction accuracy than other models. Internal and external validations showed that the nomograms combining TNM stage with LODDS+LNR were convincingly precise. Decision curve analysis suggested the nomograms performed better than AJCC 8th TNM stage in clinical practicability.

**Conclusions:**

We constructed online nomograms for cancer-specific survival and overall survival of lung adenocarcinoma patients after surgery, which may facilitate doctors to provide highly individualized therapy.

**Supplementary Information:**

The online version contains supplementary material available at 10.1186/s12885-021-08410-6.

## Background

Lung cancer is identified as the most common cancer and the leading cause of cancer-specific deaths in the United States, accounting for about 135,720 deaths in 2020 [1]. Traditionally, lung cancer is classified as non-small cell lung cancer (NSCLC) and small cell lung cancer. NSCLC constitutes 85% of all types of lung cancer cases, and the most common histological subtypes are lung adenocarcinoma (AC) and lung squamous cell carcinoma (SCC), which accounts for 60 and 15% separately [2]. Although lung AC and SCC are commonly categorized as NSCLC, a large body of experimental evidence support that these are very distinct diseases and have vastly distinct transcriptomic profiles, histopathologic, and clinicopathological features, resulting in different prognostics [3–7]. Hence, some researchers suggested abandoning the concept of NSCLC and adopting a subtype-centered tumor classification to develop more accurate diagnostic, therapeutic, and prognostic procedures [8]. Currently the prognostic researches are mostly on NSCLC [5, 9], while the prognostic models on lung AC are scarce. In addition, the prognosis for patients with NSCLC is currently estimated based on American Joint Committee on Cancer (AJCC) 8th tumor, node, metastasis (TNM) staging system, of which the N classification is based on the lymphatic region involved. This system does not take the number of dissected lymph nodes (NDLN) and number of positive lymph nodes (NPLN) into consideration. All these limitations detract from the accuracy of the prognostic estimates of lung AC.

Although NDLN and NPLN do not contribute to N classification, several studies have confirmed that higher values of NPLN, log odds of positive lymph nodes (LODDS, calculated using the formula: $\boldsymbol{LODDS}=\mathbf{\log}\frac{\boldsymbol{NPLN}+\mathbf{0.5}\mathbf{0}}{\boldsymbol{NDLN}-\boldsymbol{NPLN}+\mathbf{0.5}}$) or lymph node ratio (LNR, calculated using the formula: $\boldsymbol{LNR}=\frac{\boldsymbol{NPLN}}{\boldsymbol{NDLN}}$) were related to worse prognosis for NSCLC or several other types of malignant tumors [10–13]. However, of these node classifications, which can obtain the most precise prognosis in lung AC is unclear.

In our study, based on the Surveillance, Epidemiology, and End Results (SEER) database, we firstly aimed to comprehensively compare the prognostic efficacy of NPLN, LODDS, and LNR classification for predicting long-term survival outcomes of patients with lung AC. Secondly, according to the result of step one, we intended to establish and validate a model combining the N classification and the selected node number-based or ratio-based staging system, which can make the best use of the information of lymph node region and number of examined and positive nodes to predict the long-term post-operative cancer-specific survival and overall survival for these patients. We present the following article in accordance with the TRIPOD reporting checklist.

## Methods

### Data source and ethics statement

The SEER database collects data from 18 cancer registries of the National Cancer Institute. SEER database includes data of nearly 34.6% of US population [14]. The data was obtained with the SEER*Stat software (version 8.3.8; RRID: SCR_003293; https://seer.cancer.gov/resources/). Primary cancer histology and site were coded by the 3rd edition of the International Classification of Diseases for Oncology (ICD-O-3). The requirement for informed consent was waived as all SEER data was deidentified before release and contained no personally identifying information of patients. The study was conducted in accordance with the Declaration of Helsinki and the Harmonized Tripartite Guideline for Good Clinical Practice from the International Conference on Harmonization. No approval by the institutional review board was sought, because SEER is a public database.

### Cohort selection

A total of 25,005 patients from SEER database were selected into our study cohort. Inclusion criteria was as followed: (I) patients diagnosed with primary lung tumor between 2004 to 2016 with site codes as C34.0-C34.9; (II) positive pathologic confirmation of histologic type as adenocarcinoma (8140–8147, 8255, 8260, 8310, 8323, 8480, 8481, 8490, 8550, 8572) based on ICD-O-3 His/Behave, malignant; (III) identified as only one primary tumor; (IV) patients who underwent a radical surgery and systematic lymph node dissection with N0–2 disease. Patients were excluded who (I) aged < 18 years; (II) were diagnosed with autopsy/death certificate only; (III) were at N3/M1 disease; (IV) had received preoperative radiotherapy; (V) had received preoperative pathological examination of resected specimens or invasive mediastinal staging; (VI) survived less than 3 months after surgery; (VII) had missing information about NDLN, NPLN, TNM staging as well as survival outcomes; (VIII) had missing information about race, laterality, site, differentiation as well as marital status at diagnosis. The TNM stages of the selected patients from SEER database were updated according to the 8th edition of the AJCC criteria [15].

According to purchased/referred care delivery areas (PRCDA) region, these patients were divided into training cohort (PRCDA = East, Northern plains, and Alaska) and external validation cohort (PRCDA = Pacific coast and Southwest). The prognostic model was subjected to bootstrap internal validation in the training cohort and external validation in the external validation cohort.

### Study covariates and outcomes

Information on the baseline demographics of the patients including age at diagnosis, sex, race, PRCDA region, marital status at diagnosis was extracted. Moreover, histopathologic features of tumor including primary site, laterality, differentiation, T classification, and N classification were included. We also extracted therapeutic strategies including type of surgery, radiotherapy, chemotherapy, NDLN, and NPLN. In addition, four continuous variables (NDLN, NPLN, LODDS, and LNR) of training cohort were trichotomized via the X-tile software (version 3.6.1) based on the maximal log-rank χ^2^ value, representing the greatest group difference in survival outcome [16]. NDLN was grouped into NDLN1 (1 to 10), NDLN2 (11 to 19), and NDLN3 (≥20), while NPLN was divided into NPLN0 (0), NPLN1 (1 to 6), and NPLN2 (7 to 61). Besides, LODDS was categorized into LODDS1 (≥ − 2.26, <− 0.94), LODDS2 (≥ − 0.94, <− 0.41), and LODDS3 (≥ − 0.41, ≤1.52), while LNR was classified into LNR0 (0), LNR1 (> 0, < 0.55), LNR2 (≥ − 0.55, ≤1.00).

In this study, we chose lung cancer-specific survival (CSS) and overall survival (OS) as primary endpoints. CSS was defined as the survival months from diagnosis to lung AC-related death, while OS was defined as the survival months from diagnosis to all-cause death. The information on follow-up and prognosis of the SEER database is updated annually and the latest follow-up data was released in December 31, 2016.

### Selection of prognostic model

To simplify the prognostic models, all the continuous variables were transformed into ranked or categorical variables and displayed as count and percentages. Baseline data of patients stratified by training and external validation cohort was compared by using Mann-Whitney U test or Pearson’s χ^2^ test for ranked variables or categorical variables respectively. 1-, 3-, and 5-year CSS and OS curves were presented by the Kaplan-Meier (KM) method, and compared using a two-sided log-rank test.

In addition, a 2-step Cox proportional hazards regression analysis was performed to identify the relation between different lymph node classifications and prognosis, and the hazard ratio (HR) and a 95% confidence interval (CI) were presented. Firstly, we performed univariate Cox regression analysis to determine which variates were potential prognostic factors. Variates with statistical significance (*P* < 0.1) in the univariate Cox regression analysis were then included in multivariate Cox regression analysis. Secondly, we separately put NPLN (Model 1), LODDS (Model 2), LNR (Model 3), LODDS+LNR (Model 4) into four different multivariate Cox regression models using Backward LR method. The predictive performance of these models was assessed according to homogeneity, statistical model fit, discrimination, and accuracy [17]. The likelihood-ratio (LR) χ^2^ test was applied to evaluate homogeneity between different models. Akaike Information Criterion (AIC) was used to measure statistical model fit. Harrell concordance index (C-index) was used to test the discriminatory ability or accuracy. In addition, comparisons of prediction accuracy between the 4 models were performed by calculating the integrated discrimination improvement (IDI) and the net reclassification improvement (NRI) [18].

### Construction and validation of the nomograms

The multivariate Cox regression models for CSS and OS with the optimal predictive performance were transformed into nomograms. Bootstrapping technique was applied on the selected model for internal validation based on 1000 resamples of the training cohort. Calibration for 1-, 3-, and 5-year CSS and OS, which compares the nomogram-predicted survival with the actual survival, was evaluated using calibration curves. The predictions were supposed to fall closely to a 45-degree diagonal line if the model was with high accuracy. Decision curve analysis (DCA) was performed to compare AJCC 8th TNM stage with our nomograms. The nomograms were transformed into convenient online versions.

Statistical analyses were performed using SPSS for windows (version 24.0; RRID: SCR_002865; https://www.ibm.com/products/spss-statistics) and R software (version 4.0.2; RRID: SCR_001905; http://www.r-project.org). All tests were two-sided and *P* value < 0.05 was considered as statistically significant.

## Results

### Patients characteristics

Between January 2004 and December 2016, the SEER database collected 241,147 patients diagnosed with lung AC. After employing the selection criteria, the final study cohort included 25,005 patients. 13,551 of them were categorized as training cohort (PRCDA = East, Northern plains, and Alaska) and the external validation cohort (PRCDA = Pacific coast, Southwest) consisted of 11,454 patients. The inclusion and exclusion process were summarized in *Additional file Table* *S1*. Baseline demographic and clinicopathological features of all patients stratified by training and external validation cohort were listed in Table 1. The median [interquartile range (IQR)] diagnostic age was 67 years (60–74 years). Most patients were diagnosed in the age of ≥50 years, were diagnosed at N0 disease (74.1%), were Caucasians (81.6%), and received lobectomy (86.0%). Interestingly patients with N0 disease (18,541) were less than patients with NPLN0 disease (18,726), which may be attributed to that part of N classification in SEER database is clinical N classification assessed by imaging manifestations.

**Table 1:** Baseline characteristics of training cohort and external validation cohort

Characteristic	Total (*n* = 25,005)		Training cohort (*n* = 13,551)		External validation cohort (*n* = 11,454)		P value
	N	%	N	%	N	%	
Age at diagnosis (year)							< 0.001
< 50	1288	5.2	806	5.9	482	4.2	
50–59	4898	19.6	2967	21.9	1931	16.9	
60–69	8830	35.3	4829	35.6	4001	34.9	
70–79	7683	30.7	3894	28.7	3789	33.1	
≥ 80	2306	9.2	1055	7.8	1251	10.9	
Sex							0.021
Male	10,680	42.7	5878	43.4	4802	41.9	
Female	14,325	57.3	7673	56.6	6652	58.1	
Race							< 0.001
White	20,416	81.6	11,639	85.9	8777	76.6	
Black	2287	9.1	1638	12.1	649	5.7	
Other	2302	9.2	274	2.0	2028	17.7	
Marital status at diagnosis							< 0.001
Single	2942	11.8	1479	10.9	1463	12.8	
Married	14,917	59.7	8095	59.7	6822	59.6	
Other	7146	28.6	3977	29.3	3169	27.7	
Laterality							0.026
Right	15,015	60.0	8223	60.7	6792	59.3	
Left	9990	40.0	5328	39.3	4662	40.7	
Site							0.006
Main bronchus	40	0.2	21	0.2	19	0.2	
Upper lobe	15,359	61.4	8454	62.4	6905	60.3	
Middle lobe	1317	5.3	674	5.0	643	5.6	
Lower lobe	7983	31.9	4231	31.2	3752	32.8	
Over lapping lesion of lung	306	1.2	171	1.3	135	1.2	
Differentiation							< 0.001
I (Well differentiated)	4568	18.3	2298	17.0	2270	19.8	
II (Medium differentiated)	12,401	49.6	6655	49.1	5746	50.2	
III (Poorly differentiated)	7851	31.4	4506	33.3	3345	29.2	
IV (Undifferentiated)	185	0.7	92	0.7	93	0.8	
T classification							0.028
T1	11,856	47.4	6529	48.2	5327	46.5	
T2	8832	35.3	4701	34.7	4131	36.1	
T3	2976	11.9	1580	11.7	1396	12.2	
T4	1341	5.4	741	5.5	600	5.2	
N classification							0.502
N0	18,541	74.1	10,080	74.4	8461	73.9	
N1	3366	13.5	1774	13.1	1592	13.9	
N2	3098	12.4	1697	12.5	1401	12.2	
Type of surgery							< 0.001
Sublobectomy	2831	11.3	1707	12.6	1124	9.8	
Lobectomy	21,499	86.0	11,441	84.4	10,058	87.8	
Pneumonectomy	675	2.7	403	3.0	272	2.4	
Radiotherapy							< 0.001
No	22,786	91.1	12,262	90.5	10,524	91.9	
Yes	2219	8.9	1289	9.5	930	8.1	
Chemotherapy							< 0.001
No	18,282	73.1	9737	71.9	8545	74.6	
Yes	6723	26.9	3814	28.1	2909	25.4	
NDLN							0.277
NDLN1 (1–10)	15,958	63.8	8717	64.3	7241	63.2	
NDLN2 (11–19)	6399	25.6	3345	24.7	3054	26.7	
NDLN3 (≥20)	2648	10.6	1489	11.0	1159	10.1	
NPLN							0.301
NPLN0 (0)	18,726	74.9	10,186	75.2	8540	74.6	
NPLN1 (1–6)	5656	22.6	3022	22.3	2634	23.0	
NPLN2 (≥7)	623	2.5	343	2.5	280	2.4	
LODDS							0.371
LODDS1 (<−0.94)	15,712	62.8	8427	62.2	7285	63.6	
LODDS2 (≥ − 0.94, <−0.41)	6211	24.8	3451	25.5	2760	24.1	
LODDS3 (≥ − 0.41)	3082	12.3	1673	12.3	1409	12.3	
LNR							0.045
LNR0 (0)	18,726	74.9	10,186	75.2	8540	74.6	
LNR1 (> 0, < 0.55)	5120	20.5	2716	20.0	2404	21.0	
LNR2 (≥0.55, ≤1)	1159	4.6	649	4.8	510	4.5	

*NDLN* number of dissected lymph nodes; *NPLN* number of positive lymph nodes; *LODDS* log odds of positive lymph nodes; *LNR* lymph node ratio

### Survival analysis

**Fig. 1:**
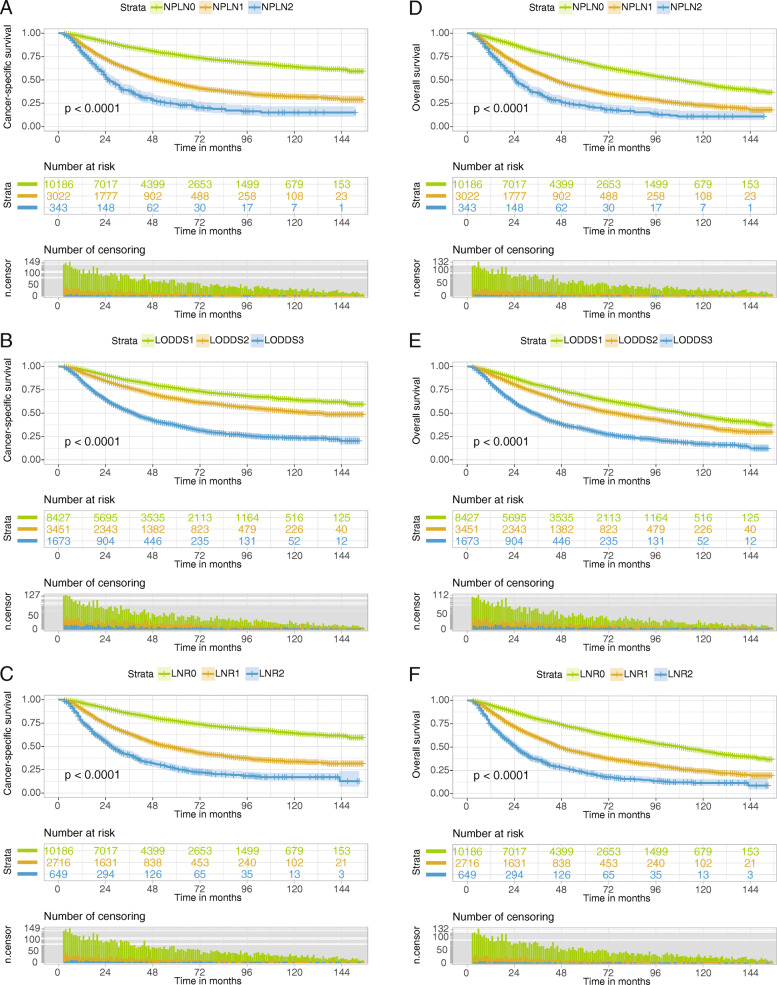
Kaplan-Meier estimates of CSS (**A**, **B**, **C**) and OS (**D**, **E**, **F**) for training cohort according to NPLN, LODDS, and LNR. CSS, cancer-specific survival; OS, overall survival; AC, adenocarcinoma; NPLN, number of positive lymph nodes; LODDS, log odds of positive lymph nodes; LNR, lymph node ratio

**Fig. 2:**
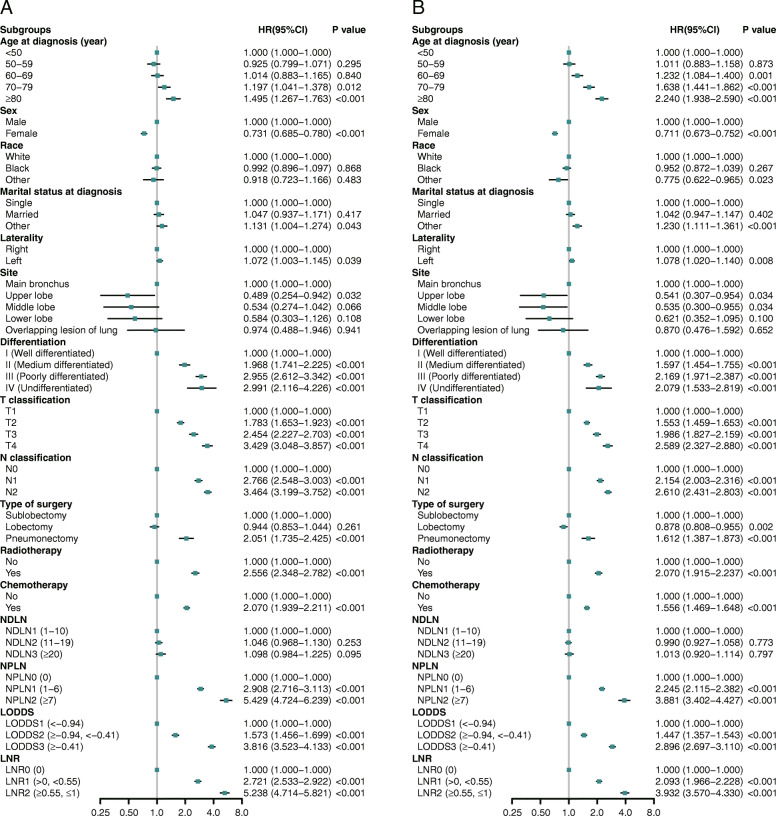
Univariable Cox regression analysis and forest plot of potential prognostic predictors for CSS (A) and OS (B) in training cohort

**Fig. 3:**
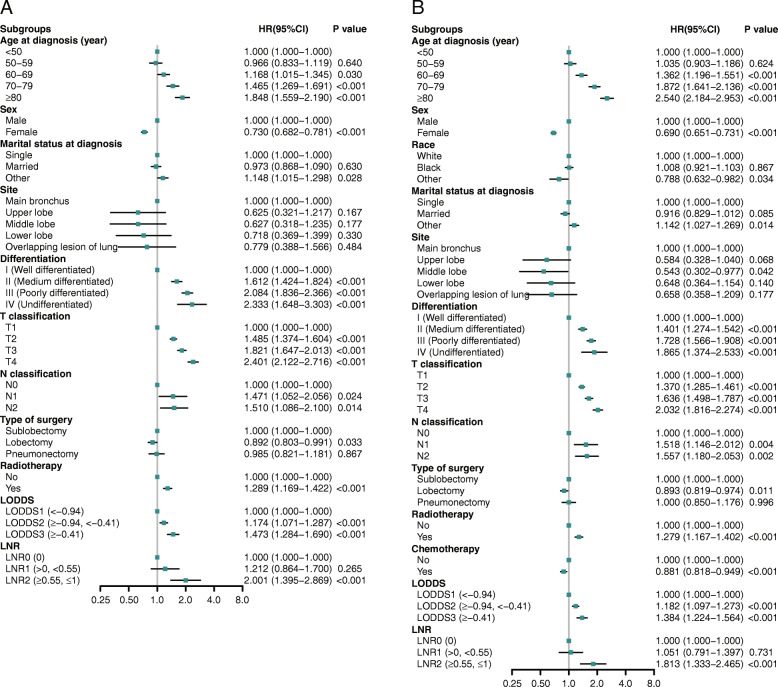
Multivariable Cox regression analysis and forest plot [Model 4 (LODDS+LNR)] of prognostic predictors for CSS (A) and OS (B) in training cohort

The median (IQR) follow-up times on all selected patients were 65 months (30–105 months) by calculating the interval of time from the median patient entry to the cutoff date for analysis. The CSS and OS of the training cohort patients stratified by different node classifications were shown in *Additional file Table* *S2* and *Additional file Table* *S3*. The cumulative 1-, 3-, 5-year CSS rates for LNR0 patients were 96.4% (95% CI, 96.0–96.8%), 85.1 (95% CI, 84.3–85.9%), and 77.0% (95% CI, 76.0–78.0%) respectively, and were 89.6% (95% CI, 88.4–90.8%), 63.3 (95% CI, 61.4–65.4%), and 48.0 (95% CI, 45.8–50.3%) for LNR1 patients respectively, and were 75.6% (95% CI, 72.3–79.1%), 40.7 (95% CI, 36.7–45.1%), and 26.0 (95% CI, 22.3–30.3%) for LNR2 patients respectively. As displayed in Fig. 1, patients with higher value of NPLN, LNR, and LODDS were significantly related with lower CSS and OS rates (log-rank test *P* < 0.001). In Fig. 2a, Univariate Cox regression analysis demonstrated that age at diagnosis, sex, marital status at diagnosis, laterality, site, differentiation, T classification, N classification, type of surgery, radiotherapy, chemotherapy, NDLN, NPLN, LNR, and LODDS showed significant relationship with CSS (*P* < 0.1). Similarly, the potential prognostic variates for OS were also displayed in Fig. 2b. Subsequently, using Backward LR method, we separately incorporated NPLN (Model 1), LODDS (Model 2), LNR (Model 3), and LODDS+LNR (Model 4) into four multivariate Cox regression models combined with the same covariates. Multivariate Cox regression analysis (Model 1–3) showed that all the three classifications (NPLN, LODDS, LNR) were independent prognostic variates for CSS and OS in *Additional file Table* *S4* and *Additional file Table* *S5*. Model 4 for CSS and OS was demonstrated in Fig. 3a and b.

### Comparison of predictive performance between NPLN, LODDS, and LNR

The predictive performances of Model 1–4 were evaluated and listed in *Table* *S6*. In Model 1–3, LNR (Model 3) showed higher LR χ^2^ test, and lower AIC than NPLN and LODDS (Model 1–2). LNR (Model 3) and LODDS (Model 2) showed similar C-indexes, which were higher than NPLN (Model 1). In other words, LNR exhibited superior predictive potential to NPLN and LODDS. In addition, LODDS+LNR (Model 4) manifested the highest LR χ^2^ test (2022.4 for nomogram for CSS, 2085.1 for nomogram for OS), highest C-index (0.7241 for nomogram for CSS, 0.6941 for nomogram for OS), and lowest AIC (63,133 for nomogram for CSS, 87,559 for nomogram for OS) in all four models. What’s more, the comparisons between LODDS+LNR (Model 4) and other models using IDI and NRI were shown in Table 2. All the IDI and NRI values were < 0 with most *P* values < 0.05, suggesting Model 4 had better predicting performance than other models.

**Table 2:** Comparison of predictive performance of different classification in training cohort

Model	IDI (95%CI)	P	NRI (95%CI)	P
Cancer-specific survival				
Model 1 (NPLN)	−0.006 (−0.010 to −0.002)	< 0.001	−0.000 (−0.054 to 0.029)	0.884
Model 2 (LODDS)	−0.006 (−0.009 to −0.003)	< 0.001	−0.119 (−0.160 to 0.016)	0.093
Model 3 (LNR)	−0.002 (−0.004 to −0.000)	< 0.001	−0.094 (−0.125 to −0.068)	< 0.001
Model 4 (LODDS+LNR)	Reference		Reference	
Overall survival				
Model 1 (NPLN)	−0.008 (−0.013 to −0.005)	< 0.001	−0.111 (−0.132 to −0.050)	< 0.001
Model 2 (LODDS)	−0.006 (−0.010 to −0.004)	< 0.001	−0.067 (−0.131 to 0.039)	0.266
Model 3 (LNR)	−0.001 (−0.003 to −0.000)	< 0.001	−0.088 (−0.136 to −0.066)	< 0.001
Model 4 (LODDS+LNR)	Reference		Reference	

*IDI* integrated discrimination improvement; *NRI*, net reclassification improvement; *NPLN* number of positive lymph nodes; *LODDS* log odds of positive lymph nodes; *LNR* lymph node ratio

### Construction and validation of nomograms

**Fig. 4:**
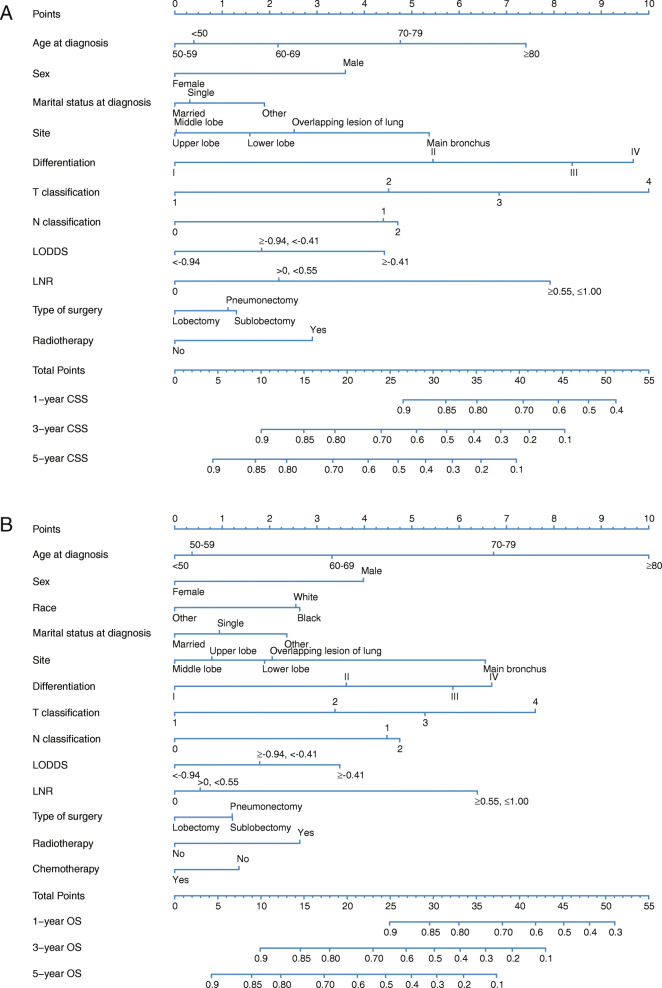
Nomograms to predict 1-, 3- and 5-year CSS (**A**) and OS (**B**) for patients with lung AC after surgery. LODDS, log odds of positive lymph nodes; LNR, lymph node ratio; CSS, cancer-specific survival; OS, overall survival; AC, adenocarcinoma

Significant independent prognostic factors, including age at diagnosis, sex, marital status at diagnosis, site, differentiation, T, N, LODDS, LNR, type of surgery, and radiotherapy were incorporated into the nomogram for CSS prediction based on Model 4 (Fig. 4a). Likewise, nomogram for OS was also established (Fig. 4b). 1-, 3-, and 5-year CSS and OS can be calculated by these nomograms. As shown in the nomogram for CSS, T classification and differentiation made the largest contribution to this nomogram, followed by LNR, age at diagnosis, site, and other variates. While in the nomogram for OS, age at diagnosis and T classification made the largest contribution, followed by differentiation, site, LNR and other variates. Each factor of these variates corresponded to a score on the point scale. The total score could be calculated by adding these points and by drawing a straight line down from the total score, the estimated CSS or OS rate could be determined.

**Fig. 5:**
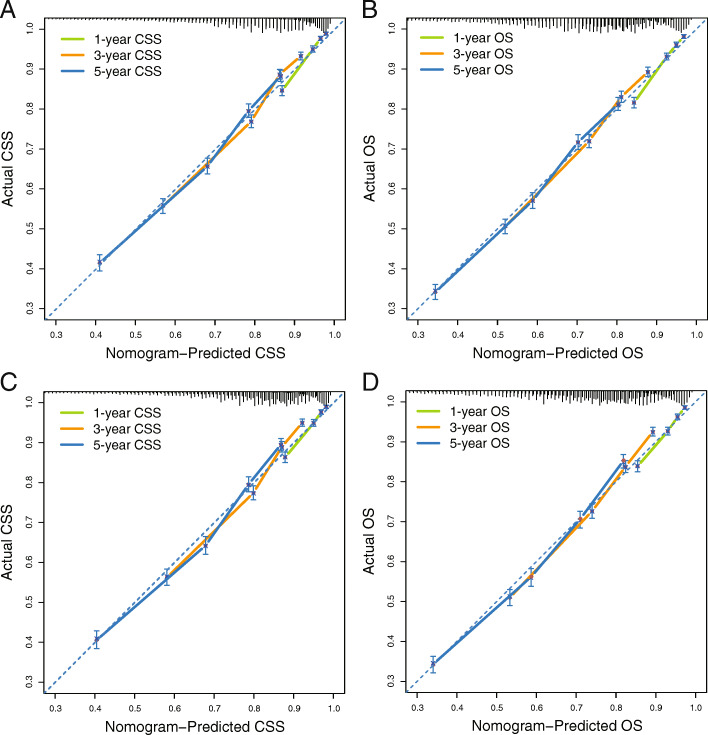
Calibration plots of the nomograms for CSS, OS prediction of the training cohort (**A**, **B**), and external validation cohort (C, D). CSS, cancer-specific survival; OS, overall survival

**Fig. 6:**
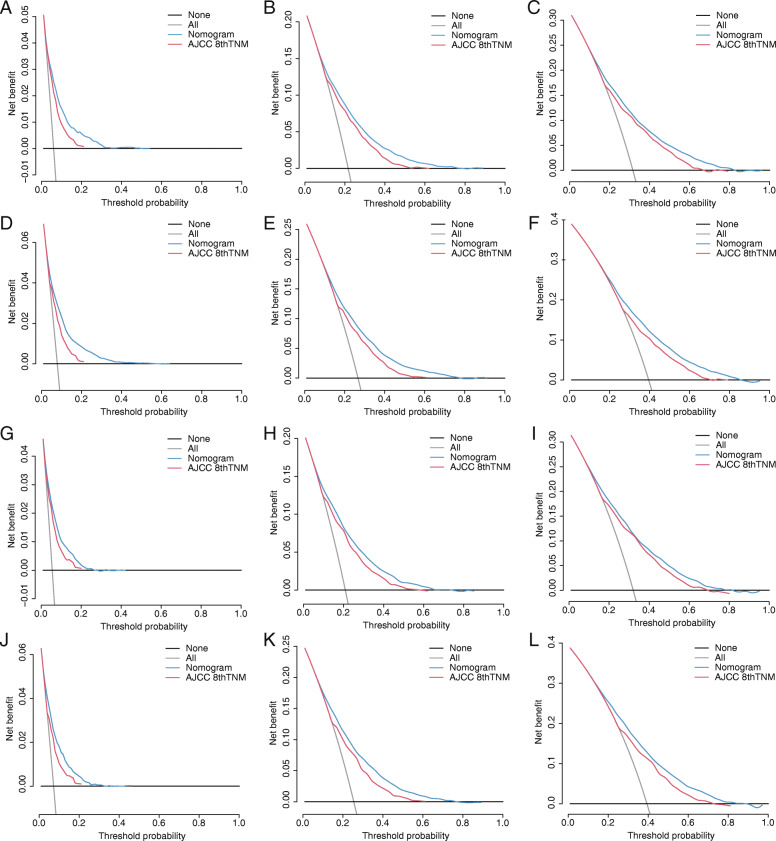
DCA of AJCC 8th TNM stage and nomogram for 1-, 3-, and 5-year CSS (**A**, **B**, **C**), OS (**D**, **E**, **F**) prediction of the training cohort; DCA of AJCC 8th TNM stage and nomogram for 1-, 3-, and 5-year CSS (G, H, I), OS (**J**, **K**, **L**) prediction of the external validation cohort. DCA, decision curve analysis; AJCC, American Joint Committee on Cancer; TNM, tumor, node, metastasis; CSS, cancer-specific survival; OS, overall survival

Internal validation was performed using bootstrapping technique based on 1000 resamples of the study cohort, and the adjusted C-indexes (0.7222 for nomogram for CSS, 0.6920 for nomogram for OS) were presented, indicating a good prediction performance. For the external validation cohort, the C-indexes (0.7302 for nomogram for CSS, 0.7060 for nomogram for OS) were also high. Besides, the calibration plots (Fig. 5) showed the points were close to the 45-degree line, indicating a good coincidence between nomogram-predicted and actual CSS or OS. For both the training cohort and validation cohort, the DCA curves (Fig. 6) for CSS and OS suggested that our nomograms were better than the AJCC 8th TNM staging system. All these results suggested that the nomograms are convincingly accurate and practical.

### Development of webservers for convenient clinical use

Two online dynamic nomograms based on Model 4 were built (https://drboidedwater.shinyapps.io/DynNom-CSS-lungadenocarcinoma/; https://drboidedwater.shinyapps.io/DynNom-OS-lungadenocarcinoma/). The calculation of LODDS and LNR can be troublesome for doctors, while in our nomograms, LODDS and LNR can be calculated automatically using NDLN and NPLN. By putting in the covariates, predicted survival probability and Kaplan-Meier curves can be generated by the webservers.

## Discussion

The current nodal status of 8th TNM staging system was based on the notion that nodal metastasis takes place first in the nodes closest to the primary tumor and diffuse to more distant nodes sequentially [19]. It categorizes metastasis to ipsilateral peribronchial and/or hilar nodes and intrapulmonary nodes as N1, and classify metastasis into ipsilateral mediastinal and/or subcarinal nodes into N2, without considering number of metastatic lymph node [20]. Surgery quality and the precision of N classification are closely related to the number of lymph node retrieval. If the surgeon cannot achieve sufficient lymph node dissection, the pathological report may lead to N classification migration, leading to insufficient therapy and worse prognosis. Likewise, the accuracy of NPLN staging system is also dependent on number of lymph node dissected and surgery quality.

The widely used N classification and NPLN have been questioned, and LODDS or LNR was believed to be superior to N classification, NPLN or NDLN considered separately for predicting survival outcomes and proposed to be applied to lymph node classification [11, 21, 22]. LODDS and LNR are ratio-based nodal evaluation methods and both include the number of dissected and positive lymph nodes to achieve a better prognostic efficacy which could overcome the limitation of number-based assessment, namely, NDLN and NPLN classification to some extent. Based on those discussions mentioned above, we undertook this study to establish an optimal multivariate Cox regression model to predict survival outcomes of lung AC patients after surgery. We found that NPLN, LODDS and LNR were all independent predictive factors, and LNR was superior to NPLN and LODDS by demonstrating better performance in homogeneity, statistical model fit, discrimination, and accuracy of survival prediction. LODDS was thought to be prognostically superior or equivalent to lymph node ratio in previous lung or digestive cancer studies [10–13, 17, 23]. Deng et al. studied the predictive performance of LODDS and LNR in patients with node-positive NSCLC and chose 10 as a cutoff value of NDLN [11]. They found that when NDLN < 10, LODDS was slightly superior to LNR and LNR performed better when NDLN ≥10. Zhao et al. performed a single center study on patients with lung AC and supposed that LODDS staging system performs better than LNR [24].

While in our study cohort, LNR (Model 3) showed slightly better prediction performance than LODDS (Model 2), which might be because of the difference of centers, race composition of population, study time window and covariates included in the model. All these studies suggest that putting these two ratio-based classifications into nodal classification for patients with lung AC would be reasonable and feasible.

To the best of our knowledge, this is the first study establishing a novel nomogram combining N, LODDS, and LNR classifications to predict postoperative long-term CSS or OS for lung AC. It is also a multicenter study with the largest population of patients with resectable lung AC. Previous studies concentrate on comparing the N, LODDS, and LNR classifications separately, while considering the importance of accurate evaluation of lung AC for optimizing and implementing of therapeutic methods, we innovatively incorporated LODDS and LNR staging system into one prognostic model (Model 4) to strengthen the TNM stage, which can take full advantage of the valuable pathological evidence retrieved from surgery. As we can see in Table 2, Model 4 exhibited the most powerful prognostic prediction in all 4 models. In this model, patients with higher level of N classification, higher value of LODDS or LNR tend to have worse CSS or OS, suggesting more aggressive therapeutic strategies and close follow-up are needed in this cohort.

As for the NDLN, a larger NDLN is closely associated with more-accurate node staging and better long-term survival outcomes and there is no final conclusion on optimal NDLN [25–27]. Liang et al. reported 16 as the cutoff value for assessing the quality of LN examination and prognostic prediction for patients with declared node-negative NSCLC [26]. While Dai et al. suggested the optimal NDLN correlated with tumor size, and the optimal NDLN for patients with T1a, T1b, T1c, and T2a NSCLC was 8, 9, 10, and 11 respectively [28]. In our study, the X-tile software trichotomized NDLN into NDLN1 (1 to 10), NDLN2 (11 to 19), and NDLN3 (≥20), and NDLN turned out to be independent prognostic factor in Model 1 for CSS. Based on comprehensive evaluation on these models, we consider patients with lung AC could benefit more from NDLN ≥11 than NDLN < 11. Our study revealed that patients who underwent radiotherapy were associated with a significantly worse CSS or OS. This may be attributed to that patients with radiotherapy were in higher TNM stage and have poor prognostic outcomes essentially. Which cohort could benefit from radiotherapy still needs further research. Another interesting finding in our study was that the long-term CSS and OS of patients with a married status at diagnosis had no significant difference from patients with a single status (never married), while the patients with other marital status at diagnosis (including divorced, separated, unmarried/domestic partner, and widow) had worse long-term CSS and OS (*Additional file Table* *S4*, *Additional file Table* *S5*, and Fig. 3). Zhang’s group demonstrated that married status was an independent protective factor for both CSS and OS for soft tissue sarcoma, and widowed patients had the highest death risk among the unmarried groups [29]. A propensity-adjusted study found that patients with NSCLC who were married had better CSS compared to unmarried ones, and among the unmarried ones, patients who were single had worse CSS than those who were divorced or widowed [30]. The different conclusion concerning influence of single status on prognosis may be due to the different TNM stage and histology of lung cancer between our studies. However, the impact of the subtypes of other marital status on long-term survival of lung AC patients need further investment.

In the end, two convincing nomograms based on Model 4 for CSS and OS were established with relatively high C-indexes, and they’re internally validated using bootstrapping technique and externally validated using calibration curves. Well-corresponded calibration plots were exhibited for the prediction of CSS and OS using the nomograms. Most importantly, a generalizable conclusion could be drawn based on our multicenter and large cohort study. The online nomograms, consisted of a few easily obtained prognostic factors, can help doctors in evaluation of death risks, patient counseling, and decision-making. In other words, patients with worse survival outcomes estimated by the nomograms may need more aggressive treatments, including chemotherapy and radiotherapy.

Our study had several limitations. First, SEER database is lacked of some potential prognostic factors, such as smoking history, sequence of surgery and chemotherapy, specific chemotherapy regimens, tyrosine kinase inhibitors treatment, immune checkpoint inhibitors treatment etc. Second, as this is a population study, we are unable to use uniform counting methods, thus may resulting into underestimation when the lymph nodes are adhesive to each other or difficult to be separated from the dissected tissues and overestimation when fragmentation of nodal tissues happens. Third, there is no recording of recurrence-free survival in the SEER database.

## Conclusions

We confirmed the value of combination of LODDS with LNR was superior to NPLN, LODDS, and LNR separately in predicting survival outcomes in lung AC patients who received surgery. Online dynamic nomograms including 8th TNM stage complemented by LODDS and LNR to evaluate CSS and OS were constructed. The well-performed nomograms may facilitate doctors to provide smarter, individualized therapy for lung AC patients.

## Supplementary Information

**Additional file 1: Table S1.** Selection procedure of study cohort. **Table S2.** 1-, 3-, and 5-year cancer-specific survival rate of subgroups of different classifications in training cohort. **Table S3.** 1-, 3-, and 5-year overall survival rate of subgroups of different classifications in training cohort. **Table S4.** Multivariable Cox regression analysis (Model 1–3) of prognostic predictors for CSS in training cohort. **Table S5.** Multivariable Cox regression analysis (Model 1–3) of prognostic predictors for OS in training cohort. **Table S6.** Comparison of predictive performance of different classifications in training cohort.

## Data Availability

The dataset supporting the conclusions of this article is available in the SEER*Stat software (version 8.3.8; RRID:SCR_003293; https://seer.cancer.gov/resources/).

## Abbreviations

AC: Adenocarcinoma
AIC: Akaike information criterion
AJCC: American Joint Committee on Cancer
CI: Confidence interval
C-index: Harrell concordance index
CSS: Cancer-specific survival
DCA: Decision curve analysis
HR: Hazard ratio
ICD-O-3: 3rd edition of the International Classification of Diseases for Oncology
IDI: Integrated discrimination improvement
IQR: Interquartile range
KM: Kaplan-Meier
LNR: Lymph node ratio
LODDS: Log odds of positive lymph nodes
LR: Likelihood ratio
NDLN: Number of dissected lymph nodes
NPLN: Number of positive lymph nodes
NRI: Net reclassification improvement
NSCLC: Non-small cell lung cancer
OS: Overall survival
PRCDA: Purchased/referred care delivery areas
SEER: Surveillance, Epidemiology, and End Results
TNM: Tumor, node, metastasis
