# Supplementary material for: Dynamic nomograms combining N classification with ratio-based nodal classifications to predict long-term survival for patients with lung adenocarcinoma after surgery: a SEER population-based study
Source: BMC Cancer. 2021 Aug 4;21:653. doi: 10.1186/s12885-021-08410-6 (PMC8336099; doi:10.1186/s12885-021-08410-6)
Supplement: Supplementary file 1 — Additional file 1: Table S1. Selection procedure of study cohort. Table S2. 1-, 3-, and 5-year cancer-specific survival rate of subgroups of different classifications in training cohort. Table S3. 1-, 3-, and 5-year overall survival rate of subgroups of different classifications in training cohort. Table S4. Multivariable Cox regression analysis (Model 1–3) of prognostic predictors for CSS in training cohort. Table S5. Multivariable Cox regression analysis (Model 1–3) of prognostic predictors for OS in training cohort. Table S6. Comparison of predictive performance of different classifications in training cohort. [file 12885_2021_8410_MOESM1_ESM.docx]

**Table S1** Selection procedure of study cohort

| Step | Criteria | Number excluded | Number remained |
| --- | --- | --- | --- |
| 1 | Patients with lung AC between 2004 and 2016 | - | 241,147 |
| 2 | Include if identified as only one primary tumor | 76,106 | 165,041 |
| 3 | Include if aged ≥18 years | 5 | 165,036 |
| 4 | Include if treated with radical surgery and systematic LN dissection | 132,535 | 32,501 |
| 5 | Exclude if diagnosed with autopsy/death certificate only | 336 | 32,165 |
| 6 | Exclude if N3 disease | 146 | 32,019 |
| 7 | Exclude if M1 disease (metastasis) | 1,595 | 30,424 |
| 8 | Exclude if receiving preoperative radiotherapy | 783 | 29,641 |
| 9 | Exclude if survival time of less than 3 month after surgery | 1,645 | 27,996 |
| 10 | Missing information about NDLN, NPLN, TNM staging as well as survival outcomes | 349 | 27,647 |
| 11 | Missing information about race, laterality, site, differentiation as well as marital status at diagnosis | 2,642 | 25,005 |

AC, adenocarcinoma; LN, lymph node; NDLN, number of dissected lymph nodes; NPLN, number of positive lymph nodes; TNM, tumor node metastasis.

**Table S2** 1-, 3-, and 5-year cancer-specific survival rate of subgroups of different classifications in training cohort

| Study cohort | 1-year survival  rate (%) | 3-year survival  rate (%) | 5-year survival  rate (%) | P value |
| --- | --- | --- | --- | --- |
| Total | 94.0 (93.6-94.4) | 78.4 (77.6-79.2) | 68.4 (67.4-69.3) |  |
| NPLN |  |  |  | <0.001 |
| NPLN0 | 96.4 (96.0-96.8) | 85.1 (84.3-85.9) | 77.0 (76.0-78.0) |  |
| NPLN1 | 87.7 (86.5-88.9) | 61.2 (59.3-63.2) | 45.9 (43.8-48.1) |  |
| NPLN2 | 79.2 (74.9-83.8) | 38.3 (33.0-44.6) | 24.1 (19.2-30.2) |  |
| LODDS |  |  |  | <0.001 |
| LODDS1 | 96.4 (96.0-96.8) | 84.8 (84.0-85.7) | 76.7 (75.6-77.8) |  |
| LODDS2 | 93.5 (92.7-94.4) | 76.8 (75.3-78.4) | 65.3 (63.4-67.2) |  |
| LODDS3 | 83.4 (81.6-85.2) | 51.6 (49.0-54.2) | 36.9 (34.3-39.7) |  |
| LNR |  |  |  | <0.001 |
| LNR0 | 96.4 (96.0-96.8) | 85.1 (84.3-85.9) | 77.0 (76.0-78.0) |  |
| LNR1 | 89.6 (88.4-90.8) | 63.3 (61.4-65.4) | 48.0 (45.8-50.3) |  |
| LNR2 | 75.6 (72.3-79.1) | 40.7 (36.7-45.1) | 26.0 (22.3-30.3) |  |

Data were presented as survival rate (%) with a 95% confidence interval (CI) in brackets; log-rank test was used to assess statistical differences between subgroups. NPLN, number of positive lymph nodes; LODDS, log odds of positive lymph nodes; LNR, lymph node ratio.

**Table S3** 1-, 3-, and 5-year overall survival rate of subgroups of different classifications in training cohort

| Study cohort | 1-year survival  rate (%) | 3-year survival  rate (%) | 5-year survival  rate (%) | P value |
| --- | --- | --- | --- | --- |
| Total | 92.2 (91.7-92.6) | 73.4 (72.6-74.2) | 60.5 (59.5-61.5) |  |
| NPLN |  |  |  | <0.001 |
| NPLN0 | 94.6 (94.1-95.0) | 79.7 (78.8-80.6) | 68.1 (67.0-69.2) |  |
| NPLN1 | 85.8 (84.5-87.1) | 57.2 (55.3-59.1) | 40.4 (38.4-42.5) |  |
| NPLN2 | 77.6 (73.2-82.3) | 36.0 (30.8-42.0) | 22.3 (17.8-28.1) |  |
| LODDS |  |  |  | <0.001 |
| LODDS1 | 94.6 (94.1-95.1) | 79.9 (78.9-80.9) | 68.4 (67.2-69.7) |  |
| LODDS2 | 91.7 (90.8-92.6) | 71.0 (69.4-72.7) | 56.4 (54.5-58.4) |  |
| LODDS3 | 81.5 (79.6-83.4) | 47.9 (45.4-50.5) | 32.8 (30.3-35.4) |  |
| LNR |  |  |  | <0.001 |
| LNR0 | 94.6 (94.1-95.0) | 79.7 (78.8-80.6) | 68.1 (67.0-69.2) |  |
| LNR1 | 87.9 (86.7-89.2) | 59.7 (57.7-61.7) | 42.7 (40.6-45.0) |  |
| LNR2 | 72.7 (69.3-76.3) | 36.3 (32.5-40.4) | 22.3 (18.9-26.2) |  |

Note: data were presented as survival rate (%) with a 95% confidence interval (CI) in brackets; log-rank test was used to assess statistical differences between subgroups. NPLN, number of positive lymph nodes; LODDS, log odds of positive lymph nodes; LNR, lymph node ratio.

**Table** **S4** Multivariable Cox regression analysis (Model 1-3) of prognostic predictors for CSS in training cohort

| Characteristic | Model 1 (NPLN) | | |  | Model 2 (LODDS) | |  | Model 3 (LNR) | |
| --- | --- | --- | --- | --- | --- | --- | --- | --- | --- |
|  | HR (95%CI) | P value | |  | HR (95%CI) | P value |  | HR (95%CI) | P value |
| Age at diagnosis (year) |  | |  |  |  |  |  |  |  |
| <50 | 1 | |  |  | 1 |  |  | 1 |  |
| 50-59 | 0.943 (0.814-1.093) | | 0.436 |  | 0.952 (0.822-1.103) | 0.511 |  | 0.967 (0.834-1.120) | 0.653 |
| 60-69 | 1.138 (0.988-1.310) | | 0.072 |  | 1.151 (1.000-1.325) | 0.050 |  | 1.166 (1.013-1.343) | 0.032 |
| 70-79 | 1.428 (1.237-1.649) | | <0.001 |  | 1.442 (1.249-1.664) | <0.001 |  | 1.463 (1.267-1.689) | <0.001 |
| ≥80 | 1.796 (1.515-2.128) | | <0.001 |  | 1.807 (1.525-2.142) | <0.001 |  | 1.857 (1.567-2.201) | <0.001 |
| Sex |  | |  |  |  |  |  |  |  |
| Male | 1 | |  |  | 1 |  |  | 1 |  |
| Female | 0.734 (0.686-0.786) | | <0.001 |  | 0.737 (0.688-0.788) | <0.001 |  | 0.729 (0.681-0.780) | <0.001 |
| Marital status at diagnosis |  | |  |  |  |  |  |  |  |
| Single | 1 | |  |  | 1 |  |  | 1 |  |
| Married | 0.967 (0.863-1.084) | | 0.567 |  | 0.968 (0.864-1.084) | 0.570 |  | 0.974 (0.869-1.091) | 0.650 |
| Other | 1.139 (1.007-1.288) | | 0.038 |  | 1.143 (1.011-1.292) | 0.033 |  | 1.149 (1.016-1.299) | 0.027 |
| Site |  | |  |  |  |  |  |  |  |
| Main bronchus | 1 | |  |  | 1 |  |  | 1 |  |
| Upper lobe | 0.596 (0.306-1.160) | | 0.128 |  | 0.598 (0.307-1.164) | 0.130 |  | 0.624 (0.320-1.214) | 0.164 |
| Middle lobe | 0.601 (0.305-1.184) | | 0.141 |  | 0.596 (0.302-1.174) | 0.135 |  | 0.637 (0.324-1.256) | 0.193 |
| Lower lobe | 0.690 (1.448-1.344) | | 0.276 |  | 0.694 (0.356-1.350) | 0.282 |  | 0.719 (0.369-1.400) | 0.332 |
| Over lapping lesion of lung | 0.737 (0.366-1.481) | | 0.391 |  | 0.759 (0.378-1.526) | 0.439 |  | 0.778 (0.387-1.564) | 0.482 |
| Differentiation |  | |  |  |  |  |  |  |  |
| I (Well differentiated) | 1 | |  |  | 1 |  |  | 1 |  |
| II (Medium differentiated) | 1.610 (1.422-1.823) | | <0.001 |  | 1.613 (1.425-1.826) | <0.001 |  | 1.620 (1.431-1.834) | <0.001 |
| III (Poorly differentiated) | 2.094 (1.845-2.377) | | <0.001 |  | 2.082 (1.834-2.364) | <0.001 |  | 2.094 (1.845-2.377) | <0.001 |
| IV (Undifferentiated) | 2.219 (1.568-3.140) | | <0.001 |  | 2.256 (1.594-3.192) | <0.001 |  | 2.285 (1.614-3.234) | <0.001 |
| T classification |  | |  |  |  |  |  |  |  |
| T1 | 1 | |  |  | 1 |  |  | 1 |  |
| T2 | 1.471 (1.362-1.589) | | <0.001 |  | 1.478 (1.368-1.597) | <0.001 |  | 1.478 (1.368-1.596) | <0.001 |
| T3 | 1.804 (1.632-1.995) | | <0.001 |  | 1.802 (1.630-1.993) | <0.001 |  | 1.813 (1.640-2.004) | <0.001 |
| T4 | 2.335 (2.062-2.643) | | <0.001 |  | 2.415 (2.134-2.732) | <0.001 |  | 2.358 (2.085-2.668) | <0.001 |
| N classification |  | |  |  |  |  |  |  |  |
| N0 | 1 | |  |  | 1 |  |  | 1 |  |
| N1 | 1.456 (1.041-2.036) | | 0.028 |  | 1.722 (1.541-1.923) | <0.001 |  | 1.450 (1.038-2.027) | 0.030 |
| N2 | 1.577 (1.134-2.194) | | 0.007 |  | 1.842 (1.634-2.078) | <0.001 |  | 1.517 (1.091-2.111) | 0.013 |
| Type of surgery |  | |  |  |  |  |  |  |  |
| Sublobectomy | 1 | |  |  | 1 |  |  | 1 |  |
| Lobectomy | 0.852 (0.768-0.945) | | 0.002 |  | 0.889 (0.801-0.987) | 0.027 |  | 0.856 (0.772-0.949) | 0.003 |
| Pneumonectomy | 0.921 (0.767-1.106) | | 0.377 |  | 0.970 (0.809-1.163) | 0.740 |  | 0.940 (0.785-1.126) | 0.501 |
| Radiotherapy |  | |  |  |  |  |  |  |  |
| No | 1 | |  |  | 1 |  |  | 1 |  |
| Yes | 1.330 (1.207-1.466) | | <0.001 |  | 1.308 (1.187-1.442) | <0.001 |  | 1.308 (1.186-1.443) | <0.001 |
| NDLN |  | |  |  |  |  |  |  |  |
| NDLN1 (1-10) | 1 | |  |  |  |  |  |  |  |
| NDLN2 (11-19) | 0.871 (0.804-0.944) | | <0.001 |  |  |  |  |  |  |
| NDLN3 (≥20) | 0.745 (0.664-0.835) | | <0.001 |  |  |  |  |  |  |
| NPLN |  | |  |  |  |  |  |  |  |
| NPLN0 (0) | 1 | |  |  |  |  |  |  |  |
| NPLN1 (1-6) | 1.604 (1.154-2.229) | | 0.005 |  |  |  |  |  |  |
| NPLN2 (≥7) | 2.879 (2.024-4.094) | | <0.001 |  |  |  |  |  |  |
| LODDS |  | |  |  |  |  |  |  |  |
| LODDS1 (<-0.94) |  | |  |  | 1 |  |  |  |  |
| LODDS2 (≥-0.94, <-0.41) |  | |  |  | 1.181 (1.079-1.292) | <0.001 |  |  |  |
| LODDS3 (≥-0.41) |  | |  |  | 1.792 (1.586-2.025) | <0.001 |  |  |  |
| LNR |  | |  |  |  |  |  |  |  |
| LNR0 (0) |  | |  |  |  |  |  | 1 |  |
| LNR1 (>0, <0.55) |  | |  |  |  |  |  | 1.488 (1.069-2.069) | 0.018 |
| LNR2 (≥0.55, ≤1) |  | |  |  |  |  |  | 2.826 (2.016-3.963) | <0.001 |

CSS, cancer-specific survival; HR, hazard ratio, CI, confidence interval; NDLN, number of dissected lymph nodes; NPLN, number of positive lymph nodes; LODDS, log odds of positive lymph nodes; LNR, lymph node ratio.

**Table S5** Multivariable Cox regression analysis (Model 1-3) of prognostic predictors for OS in training cohort

| Characteristic | Model 1 (NPLN) | | |  | Model 2 (LODDS) | |  | Model 3 (LNR) | |
| --- | --- | --- | --- | --- | --- | --- | --- | --- | --- |
|  | HR (95%CI) | P value | |  | HR (95%CI) | P value |  | HR (95%CI) | P value |
| Age at diagnosis (year) |  | |  |  |  |  |  |  |  |
| <50 | 1 | |  |  | 1 |  |  | 1 |  |
| 50-59 | 0.016 (0.886-1.164) | | 0.824 |  | 1.025 (0.894-1.175) | 0.723 |  | 1.036 (0.903-1.187) | 0.615 |
| 60-69 | 1.325 (1.163-1.508) | | <0.001 |  | 1.347 (1.183-1.534) | <0.001 |  | 1.360 (1.194-1.549) | <0.001 |
| 70-79 | 1.826 (1.601-2.082) | | <0.001 |  | 1.849 (1.621-2.108) | <0.001 |  | 1.870 (1.639-2.134) | <0.001 |
| ≥80 | 2.474 (2.129-2.874) | | <0.001 |  | 2.497 (2.149-2.902) | <0.001 |  | 2.554 (2.196-2.969) | <0.001 |
| Sex |  | |  |  |  |  |  |  |  |
| Male | 1 | |  |  | 1 |  |  | 1 |  |
| Female | 0.694 (0.655-0.735) | | <0.001 |  | 0.696 (0.657-0.737) | <0.001 |  | 0.690 (0.651-0.730) | <0.001 |
| Race |  | |  |  |  |  |  |  |  |
| White |  | |  |  |  |  |  | 1 |  |
| Black |  | |  |  |  |  |  | 1.009 (0.922-1.105) | 0.842 |
| Other |  | |  |  |  |  |  | 0.789 (0.633-0.984) | 0.035 |
| Marital status at diagnosis |  | |  |  |  |  |  |  |  |
| Single | 1 | |  |  | 1 |  |  | 1 |  |
| Married | 0.907 (0.822-1.000) | | 0.050 |  | 0.910 (0.826-1.004) | 0.060 |  | 1.870 (1.639-2.134) | 0.089 |
| Other | 1.134 (1.021-1.259) | | 0.018 |  | 1.136 (1.023-1.262) | 0.017 |  | 2.554 (2.196-2.969) | 0.013 |
| Site |  | |  |  |  |  |  |  |  |
| Main bronchus | 1 | |  |  | 1 |  |  | 1 |  |
| Upper lobe | 0.534 (0.300-0.949) | | 0.033 |  | 0.559 (0.314-0.995) | 0.048 |  | 0.584 (0.328-1.040) | 0.068 |
| Middle lobe | 0.504 (0.280-0.906) | | 0.022 |  | 0.517 (0.287-0.930) | 0.028 |  | 0.554 (0.308-0.997) | 0.049 |
| Lower lobe | 0.598 (0.336-1.065) | | 0.081 |  | 0.625 (0.351-1.112) | 0.110 |  | 0.650 (0.365-1.158) | 0.144 |
| Over lapping lesion of lung | 0.590 (0.321-1.085) | | 0.089 |  | 0.634 (0.345-1.165) | 0.142 |  | 0.658 (0.358-1.211) | 0.179 |
| Differentiation |  | |  |  |  |  |  |  |  |
| I (Well differentiated) | 1 | |  |  | 1 |  |  | 1 |  |
| II (Medium differentiated) | 1.409 (1.281-1.550) | | <0.001 |  | 1.402 (1.275-1.542) | <0.001 |  | 1.408 (1.280-1.549) | <0.001 |
| III (Poorly differentiated) | 1.742 (1.578-1.923) | | <0.001 |  | 1.732 (1.569-1.912) | <0.001 |  | 1.737 (1.573-1.917) | <0.001 |
| IV (Undifferentiated) | 1.762 (1.298-2.393) | | <0.001 |  | 1.809 (1.333-2.456) | <0.001 |  | 1.823 (1.342-2.475) | <0.001 |
| T classification |  | |  |  |  |  |  |  |  |
| T1 | 1 | |  |  | 1 |  |  | 1 |  |
| T2 | 1.353 (1.269-1.442) | | <0.001 |  | 1.364 (1.280-1.455) | <0.001 |  | 1.363 (1.278-1.453) | <0.001 |
| T3 | 1.604 (1.469-1.752) | | <0.001 |  | 1.619 (1.482-1.768) | <0.001 |  | 1.627 (1.490-1.777) | <0.001 |
| T4 | 1.962 (1.753-2.196) | | <0.001 |  | 2.046 (1.829-2.290) | <0.001 |  | 2.002 (1.789-2.240) | <0.001 |
| N classification |  | |  |  |  |  |  |  |  |
| N0 | 1 | |  |  | 1 |  |  | 1 |  |
| N1 | 1.471 (1.109-1.949) | | 0.007 |  | 1.559 (1.411-1.722) | <0.001 |  | 1.504 (1.135-1.993) | 0.005 |
| N2 | 1.585 (1.202-2.090) | | 0.001 |  | 1.677 (1.501-1.872) | <0.001 |  | 1.563 (1.185-2.062) | 0.002 |
| Type of surgery |  | |  |  |  |  |  |  |  |
| Sublobectomy | 1 | |  |  | 1 |  |  | 1 |  |
| Lobectomy | 0.826 (0.759-0.899) | | <0.001 |  | 0.886 (0.813-0.966) | 0.006 |  | 0.855 (0.785-0.931) | <0.001 |
| Pneumonectomy | 0.878 (0.747-1.033) | | 0.117 |  | 0.980 (0.833-1.153) | 0.810 |  | 0.955 (0.813-1.122) | 0.573 |
| Radiotherapy |  | |  |  |  |  |  |  |  |
| No | 1 | |  |  | 1 |  |  | 1 |  |
| Yes | 1.327 (1.211-1.453) | | <0.001 |  | 1.296 (1.183-1.419) | <0.001 |  | 1.294 (1.181-1.418) | <0.001 |
| Chemotherapy |  | |  |  |  |  |  |  |  |
| No | 1 | |  |  | 1 |  |  | 1 |  |
| Yes | 0.873 (0.810-0.940) | | <0.001 |  | 0.879 (0.816-0.946) | <0.001 |  | 0.882 (0.819-0.950) | <0.001 |
| NPLN |  | |  |  |  |  |  |  |  |
| NPLN0 (0) | 1 | |  |  |  |  |  |  |  |
| NPLN1 (1-6) | 1.370 (1.040-1.806) | | 0.025 |  |  |  |  |  |  |
| NPLN2 (≥7) | 2.199 (1.629-2.969) | | <0.001 |  |  |  |  |  |  |
| LODDS |  | |  |  |  |  |  |  |  |
| LODDS1 (<-0.94) |  | |  |  | 1 |  |  |  |  |
| LODDS2 (≥-0.94, <-0.41) |  | |  |  | 1.180 (1.096-1.270) | <0.001 |  |  |  |
| LODDS3 (≥-0.41) |  | |  |  | 1.684 (1.511-1.876) | <0.001 |  |  |  |
| LNR |  | |  |  |  |  |  |  |  |
| LNR0 (0) | 1 | |  |  |  |  |  | 1 |  |
| LNR1 (>0, <0.55) |  | |  |  |  |  |  | 1.254 (0.951-1.655) | 0.109 |
| LNR2 (≥0.55, ≤1) |  | |  |  |  |  |  | 2.401 (1.804-3.194) | <0.001 |

OS, overall survival; HR, hazard ratio, CI, confidence interval; NPLN, number of positive lymph nodes; LODDS, log odds of positive lymph nodes; LNR, lymph node ratio.

**Table S6** Comparison of predictive performance of different classifications in training cohort

| Model | AIC | LR χ^2^ test | C-index |
| --- | --- | --- | --- |
| Cancer-specific survival |  |  |  |
| Model 1 (NPLN) | 63,203 | 1,951.8 | 0.7222 |
| Model 2 (LODDS) | 63,186 | 1,965.3 | 0.7231 |
| Model 3 (LNR) | 63,159 | 1,991.7 | 0.7228 |
| Model 4 (LODDS+LNR) | 63,133 | 2,022.4 | 0.7241 |
| Overall survival |  |  |  |
| Model 1 (NPLN) | 87,674 | 2,003.6 | 0.6904 |
| Model 2 (LODDS) | 87,630 | 2,010.1 | 0.6926 |
| Model 3 (LNR) | 87,586 | 2,054.2 | 0.6927 |
| Model 4 (LODDS+LNR) | 87,559 | 2,085.1 | 0.6941 |

AIC, Akaike information criterion; LR, likelihood ratio; C-index, Harrell concordance index; NPLN, number of positive lymph nodes; LODDS, log odds of positive lymph nodes; LNR, lymph node ratio.
